# Enteral Nutrition Tolerance And REspiratory Support (ENTARES) Study in preterm infants: study protocol for a randomized controlled trial

**DOI:** 10.1186/s13063-018-3119-0

**Published:** 2019-01-18

**Authors:** Francesco Cresi, Elena Maggiora, Silvia Maria Borgione, Elena Spada, Alessandra Coscia, Enrico Bertino, Fabio Meneghin, Luigi Tommaso Corvaglia, Maria Luisa Ventura, Gianluca Lista, ENTARES Study Research Group, Fabio Mosca, Anna Orsi, Domenica Mercadante, Stefano Martinelli, Laura Ilardi, Alice Proto, Sara Gatto, Arianna Aceti, Fabrizio Sandri, Roksana Chakrokh, Nicola Laforgia, Antonio Di Mauro, Maria E. Baldassarre, Antonio Del Vecchio, Flavia Petrillo, Maria P. Spalierno, Francesco Raimondi, Letizia Capasso, Marta Palma, Daniele Farina, Maria F. Campagnoli, Tatiana Boetti, Federica Logrippo, Massimo Agosti, Laura Morlacchi, Simona Perniciaro, Carlo Dani, Serena Elia, Giovanni Vento, Luca Maggio, Mauro Stronati, Elisa Civardi, Grappone Lidia, Borrelli Angela

**Affiliations:** 1 Neonatal Pathology and Neonatal Intensive Care Unit, Università di Torino, Turin, Italy; 2 Neonatal Pathology and Neonatal Intensive Care Unit, Vittore-Buzzi Children Hospital, Milan, Italy; 3 Neonatology and Neonatal Intensive Care Unit, Department of Medical and Surgical Sciences (DIMEC), University of Bologna, S.Orsola-Malpighi Hospital, Bologna, Italy; 4 Neonatal Intensive Care Unit, Fondazione MBBM, S. Gerardo Hospital, Monza, Italy

**Keywords:** Feeding intolerance, RDS, HFNC, NCPAP, Preterm, NEC, Enteral nutrition, Non-invasive ventilation, Very low birth weight infant

## Abstract

**Background:**

Respiratory distress syndrome (RDS) and feeding intolerance are common conditions in preterm infants and among the major causes of neonatal mortality and morbidity.

For many years, preterm infants with RDS have been treated with mechanical ventilation, increasing risks of acute lung injury and bronchopulmonary dysplasia.

In recent years non-invasive ventilation techniques have been developed. Showing similar efficacy and risk of bronchopulmonary dysplasia, nasal continuous positive airway pressure (NCPAP) and heated humidified high-flow nasal cannula (HHHFNC) have become the most widespread techniques in neonatal intensive care units. However, their impact on nutrition, particularly on feeding tolerance and risk of complications, is still unknown in preterm infants.

The aim of the study is to evaluate the impact of NCPAP vs HHHFNC on enteral feeding and to identify the most suitable technique for preterm infants with RDS.

**Methods:**

A multicenter randomized single-blind controlled trial was designed. All preterm infants with a gestational age of 25–29 weeks treated with NCPAP or HHHFNC for RDS and demonstrating stability for at least 48 h along with the compliance with inclusion criteria (age less than 7 days, need for non-invasive respiratory support, suitability to start enteral feeding) will be enrolled in the study and randomized to the NCPAP or HHHFNC arm. All patients will be monitored until discharge, and data will be analyzed according to an intention-to-treat model.

The primary outcome is the time to reach full enteral feeding, while parameters of respiratory support, feeding tolerance, and overall health status will be evaluated as secondary outcomes. The sample size was calculated at 141 patients per arm.

**Discussion:**

The identification of the most suitable technique (NCPAP vs HHHFNC) for preterm infants with feeding intolerance could reduce gastrointestinal complications, improve growth, and reduce hospital length of stay, thus improving clinical outcomes and reducing health costs. The evaluation of the timing of oral feeding could be useful in understanding the influence that these techniques could have on the development of sucking-swallow coordination. Moreover, the evaluation of the response to NCPAP and HHHFNC could clarify their efficacy as a treatment for RDS in extremely preterm infants.

**Trial registration:**

ClinicalTrials.gov, NCT03548324. Registered on 7 June 2018.

**Electronic supplementary material:**

The online version of this article (10.1186/s13063-018-3119-0) contains supplementary material, which is available to authorized users.

## Background

Respiratory distress syndrome (RDS) is a common condition in premature infants and one of the major causes of neonatal mortality [1]. For many years, preterm infants with RDS have been treated with mechanical ventilation, increasing the risks of acute lung injury and long-term morbidity, such as bronchopulmonary dysplasia (BPD) [2–5]. Offering an appropriate respiratory support in the delivery room, together with early surfactant administration, can allow one to avoid or limit endotracheal ventilation with better outcomes in terms of mortality and short- and long-term complications, above all BPD [6]. Early nasal continuous positive airway pressure (NCPAP) treatment combined with surfactant replacement therapy decreases the need for mechanical ventilation and has been recommended as the first line treatment for RDS [7, 8]. However, NCPAP has significant limitations, mainly related to the type of interface needed. Excessive leaks around the prongs or mask and through the mouth can lead to inadequate support, whereas excessive pressure may result in pneumothorax and damage to the nose and face. Moreover, the bulky fixation devices obscure the infant’s face, interfering with both feeding and positioning [9]. In recent years, heated humidified high-flow nasal cannula (HHHFNC) has been studied as an alternative non-invasive respiratory support (NRS). HHHFNC became popular partially due to some perceived advantages related to the type of interface used. Cannulas are easier to apply than NCPAP prongs or mask, may be more comfortable for infants, and may enable easier access to babies’ faces, thus facilitating feeding and parental bonding [10–12]. Whereas the practical advantages seem to be established, there is controversy about HHHFNC efficacy as respiratory support [13–15]. Recent studies support that HHHFNC is as effective as NCPAP for the primary treatment of RDS, but evidences are still insufficient and data are still lacking, especially for the extremely preterm population (< 28 weeks’ gestation) [14, 16–18]. A recent Cochrane review comparing HHHFNC with other NRS measures showed equivalent rates of treatment failure and similar rates of BPD when used as a post-extubation support in preterm infants [19]. With equivalent effectiveness, the choice of the most adequate NRS should consider the impact on the health status of the premature infant, evaluating above all the effect on nutrition and growth. Along with RDS, feeding intolerance (FI) represents a relevant issue in preterm infants, and the coexistence of the two represents a great challenge for the neonatologist [20]. Because of gastrointestinal immaturity, a considerable proportion of premature infants will develop clinical symptoms of FI, causing interruptions of feeding. This delays the establishment of adequate enteral nutrition and prolongs the need for parenteral nutrition, thus increasing the risk of infections and prolonging hospital stay [21]. Avoiding FI and its complications, such as necrotizing enterocolitis (NEC), is a priority for the neonatologist, who often faces the challenge of interpreting the clinical and prognostic significance of common and aspecific signs of FI. Clear identification of the parameters that should be evaluated to identify FI is still lacking in the literature, although, among controversy, the presence of gastric residuals, vomits and/or regurgitations, and abdominal distension and the onset of crises of apnea/bradycardia are considered the most frequent signs [22, 23]. A correlation between non-invasive ventilation and the occurrence of FI and NEC is plausible, although the mechanisms through which ventilation may induce FI and its incidence in ventilated infants are still unclear [20, 24]. The most common hypothesis is that pressurized gases that are not completely conveyed to the airways could cause bowel distension. Bowel distension in infants on CPAP was described by Jaile et al. [25] as *CPAP belly syndrome*, but no inferences about feeding tolerance and risk of NEC were drawn. More recent studies evaluated the effect of CPAP on mesenteric flow and gastric emptying, suggesting a role of CPAP as a risk factor for FI [26–28]. No specific studies have been designed to evaluate the impact of different types of NRS on FI and the occurrence of NEC, which are generally evaluated as secondary outcomes, susceptible to data analysis and patient selection biases. Our hypothesis is that different techniques of NRS may have different impacts on feeding issues in preterm infants.

We therefore intend to compare the application of NCPAP and HHHFNC in preterm infants with RDS to evaluate their impact on FI.

## Methods

### Aims

The aims of the study are to evaluate the effects of different NRS techniques (NCPAP vs HHHFNC) on feeding tolerance in preterm infants with RDS and to evaluate their impact on full enteral feeding (FEF) achievement and acquisition of oral feeding. A further aim is to evaluate the response to NCPAP and HHHFNC as treatment for RDS in extremely preterm infants.

### Study design and setting

The study has been designed as a multicenter randomized no-mask controlled trial. It will involve the major Italian neonatal intensive care units (NICUs) and will be coordinated by the NICU of the University of Turin.

### Inclusion criteria

All infants admitted to the NICUs with a gestational age between 25 and 29 weeks and who will have met the following inclusion criteria will be consecutively enrolled in the study:

1. Presence of RDS
2. Period of stability on HHHFNC or NCPAP for at least 48 h in the first 5 days of life (SatO_2 TC_ 90–95%, pCO_2_ ≤ 60 mmHg, FiO_2_ < 40%, Silverman score [29] ≤ 6, ≤ 2 apnea episodes/h with CPAP ≤7 cmH_2_O if on NCPAP, and flow ≤7 L/min if on HHHFNC)
3. ≤ 7 days of life
4. Suitability to start enteral feeding (if already started it should be less than 75 mL/Kg/day)
5. Parental written consent

### Exclusion criteria

The following are the study exclusion criteria:

1. Neurological or surgical diseases
2. Sepsis
3. Chromosomal abnormalities
4. Major malformations

### Recruitment and randomization

Informed written consent will be signed by both parents, and sufficient time will be allowed for consent. Non-Italian-speaking parents will only be asked for their consent if an adult interpreter is available. Trust interpreter and link worker services will be used to support involvement of participants whose first language is not Italian.

Eligible patients will be allocated to one of the two arms (NCPAP or HHHFNC) by block randomization. A software has been designed to automatically generate a randomization code and to obtain, in each research unit, a balance between patients with gestational age < 28 weeks and ≥ 28 weeks in both arms. The randomization software will be available for all research units, on a password-protected platform on the Enteral Nutrition Tolerance And REspiratory Support (ENTARES) website, and will generate a randomization sequence to which all clinicians are blind.

### Monitoring and data collection

Each research unit will adopt its own protocols for clinical management of the patients enrolled in the study while still respecting some minimal standard criteria for respiratory support and enteral nutrition, common for all participating units and defined as follows.

#### Minimal standard criteria for respiratory support

The suggested initial setup is [30, 31]:

- CPAP between 5 and 7 cmH_2_O if on NCPAP and flow between 4 and 7 L/min if on HHHFNC
- FiO_2_ set to reach pO_2_ = 50–60 mmHg (capillary/arterial blood gas test) and SatO_2_ TC = 90–95%

The criteria to try weaning are [30, 31]:

- CPAP < 4 cmH_2_O if on NCPAP and flow < 2 L/min if on HHHFNC
- FiO_2_ < 25% to maintain pO_2_ = 50–60 mmHg (capillary/arterial blood gas test) and SatO_2 TC_ = 90–95%

The failure criteria are [30, 31]:

- FiO_2_ > 40%
- pH < 7.2
- pCO_2_ > 65 mmHg
- ≥ 3 episodes of desaturations (SatO_2 TC_ ≤ 80%) per hour
- ≥ 3 episodes of apnea (> 20 s) and/or bradycardia (FC ≤ 80 beats per minute (bpm)) per hour
- Silverman score [29] > 6

#### Minimal standard criteria for enteral nutrition

The decision to increase volume of feeds will be up to the clinicians and in accordance with the protocol used in their own NICU; however, a maximum cut-off for feeding progression was set at 30 mL/kg/day [32, 33].

The indications for the interruption of feeding are based on abdominal examination, the occurrence of vomits/regurgitations and cardiorespiratory events, and the evaluation of alvus and gastric residual volumes (evaluated if required by the protocol in use) as detailed in Table 1 [22, 34, 35]. A score system was developed to evaluate abdominal distension (Table 2).

**Table 1:** Criteria for the interruption of enteral feeding

	Minor criteria	Major criteria
Physical examination	• Abdominal distension • Visible bowel ansa • Abdominal distension responsive to gastric suction/rectal stimulation	• Dyschromic abdominal wall • Abdominal distension not responsive to gastric detension/rectal stimulation • Painful abdomen
Regurgitations/vomits	• ≤ 2 episodes between 2 feeds or in the previous 3 h (if not fed)	• > 2 episodes between 2 feeds or in the previous 3 h (if not fed) • Bilious vomiting/hematemesis
Gastric residual volumes (GRVs)^a^	• GRV < 100% of previous feed (bilious or with hematic fragments)	• Hematic/fecaloidal GRV • GRV ≥ 100% of previous feed
Alvus	• Mucous stools	• Hematic stools
Cardiorespiratory (CR) events	• ≥ 3 CR events^b^/h	• ≥ 1 extreme CR event^c^
0–1 minor criteria:	- Continue enteral feeding with increments as per protocol (max 30 mL/kg/day)	
2 minor criteria:	- Stop increasing feeds, re-assess prior to the next feed, and evaluate GRV if not done before - If 2 minor criteria in at least 2 consecutive evaluations, consider reducing volume of feed	
1 major criterion or 3 minor criteria:	- Interrupt enteral feeding and re-assess prior to the next feed	

^a^The evaluation of GRVs is elective and according to the protocol of each research unit. GRVs are considered pathological according to minor and major criteria

^b^CR events were defined as episodes of apnea lasting more than 20 s or more than 5 s if followed by desaturation or bradycardia, episodes of desaturation with blood oxygen saturation below 80%, and episodes of bradycardia with heart rate below 80 beats per minute

^c^Extreme CR events were defined as CR events requiring resuscitation

**Table 2:** Abdominal distension score

Extent of distension	Score
Abdomen is not distended	0
Abdomen is distended but not tense	1
Abdomen is distended and tense, responsive to gastric suction/rectal stimulation	2
Abdomen is distended and tense, not responsive to gastric detension/rectal stimulation	3

Data on respiratory support, nutrition, growth, and overall clinical status will be collected from enrollment to discharge. According to an intention-to-treat model, each patient will be monitored whatever the occurring clinical events, including the failure of the modality of respiratory support assigned at enrollment. Death or transfer to another hospital before reaching FEF will be the only reasons for a patient to drop the study.

All data to be collected will be obtained from the clinical records. Data will be recorded on a common database available on the ENTARES website and specifically designed for this study. Access to the database will be password protected, and data will be entered by the local principal investigator. Participants will be identified by trial number only.

All data recorded throughout the study period are listed in Table 3.

**Table 3:** Data recorded during the study period

Ventilation/respiration parameters^a^	
– Respiratory support technique – PEEP/CPAP (cmH_2_O) – Peak inspiratory pressure (PIP, cmH_2_O) – Flow (L/min) – FiO_2_ (%) – Respiratory rate (acts/min) – Transcutaneous O_2_ blood saturation (SatO_2 TC_ %) – Capillary/arterial blood gas test – Episodes of apnea (> 20 s or > 5 s if followed by bradycardia/desaturation), bradycardia (Heart rate ≤ 80 bpm) and desaturation (SatO_2 TC_ ≤ 80%)/day – Silverman score	
Feeding parameters^b^	
– Parenteral nutrition intake (mL/kg/day) – Enteral nutrition intake (mL/kg/day) – Total caloric intake (Kcal/kg/day) – Type of milk: human milk or formula – Modality of feeding (bolus, gavage, continuous feeding) – Modality of fortification (if any; type of fortifier: standard, target, or adjustable fortification) – Enteral feeding interruptions (episodes/day) – Not given feeds (episodes/day) – Pathologic gastric residual volumes (episodes/day) – Vomits and/or regurgitations (episodes/day) – Abdominal distention (medium score/day)	
Auxological parameters^c^	
– Weight (g) – Length (cm) – Cranial circumference (cm)	
Overall health status parameters^d^	
– Patent ductus arteriosus – Intraventricular hemorrhage – Leukomalacia – Retinopathy of prematurity – Pneumothorax – Blood transfusion – NEC – Intestinal perforation	

^a^Ventilation/respiration parameters will be recorded at enrollment, at achievement of half enteral feeding and full enteral feeding, at the beginning of oral feeding, at achievement of full oral feeding, and at any change in respiratory assistance strategy. Apnea monitoring will extend until any respiratory support is needed (except for O_2_ supplementation per nasal cannula)

^b^Feeding parameters will be recorded daily until full enteral feeding is achieved, at the beginning of oral feeding, at achievement of full oral feeding, and at any change in respiratory assistance strategy

^c^Auxological parameters will be recorded at the time of enrollment, upon achieving half enteral feeding and full enteral feeding, and at discharge

^d^Relevant clinical events/diagnosis will be recorded from enrollment until discharge

### Outcomes

The primary outcome of the study is the time needed to reach FEF, defined as an enteral intake of 150 mL/Kg/day. Secondary outcomes are listed in Table 4.

**Table 4:** Secondary outcomes

– Time to reach HEF, defined as an enteral intake of 75 mL/Kg/day (days) – Interruptions of enteral feeding (episodes/day) – Not given feeds (episodes/day) – Pathologic gastric residual volumes (episodes/day) – Vomits and/or regurgitations (episodes/day) – Abdominal distention (mean score/day) – Beginning of oral feeding (post-menstrual age) – Time to reach full oral feeding (number of days) – Post-menstrual age at full oral feeding (weeks) – Weight growth (∆ z-score) – Duration of the respiratory support assigned at randomization (days) – Total duration of respiratory support need (days) – Failure of the respiratory support assigned at randomization (yes/no) – Length of hospital stay (days) – Duration of central venous catheter (days) – Clinical events and complications (NEC, bowel perforation, pneumothorax, BPD, PDA, ROP, IVH, PVL) – Transfers to other hospitals or deaths before reaching full enteral feeding (number of patients)	

*HEF* half enteral feeding, *NEC* necrotizing enterocolitis, *BPD* bronchopulmonary dysplasia, *PDA* patent ductus arteriosus, *ROP* retinopathy of prematurity, *IVH* intraventricular hemorrhage, *PVL* periventricular leukomalacia

**Fig. 1:**
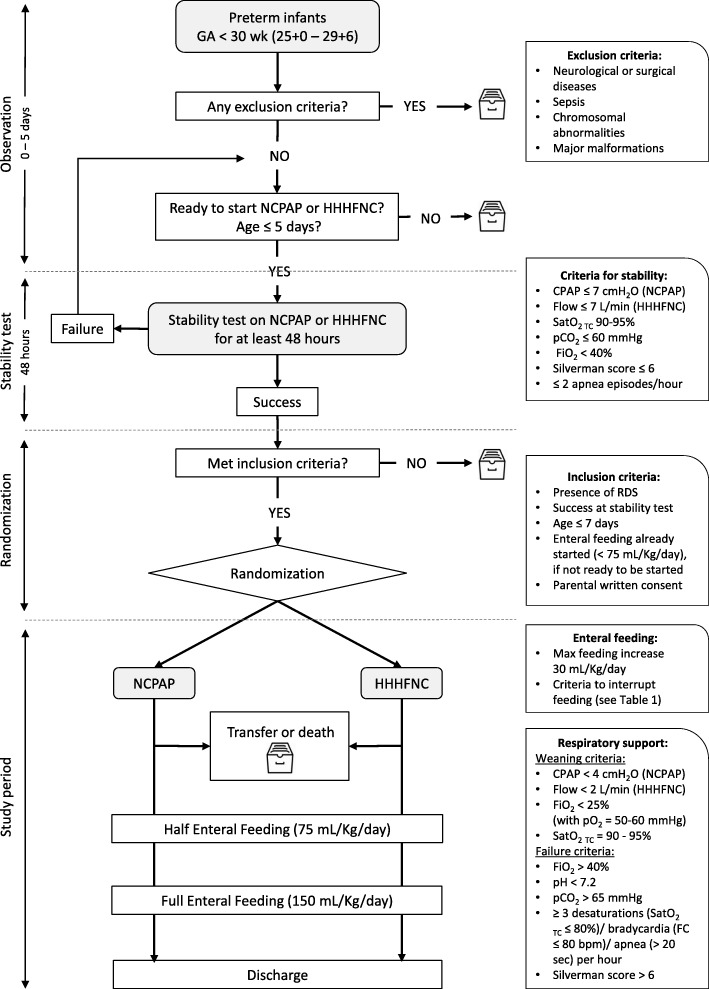
Design of the study

**Fig. 2:**
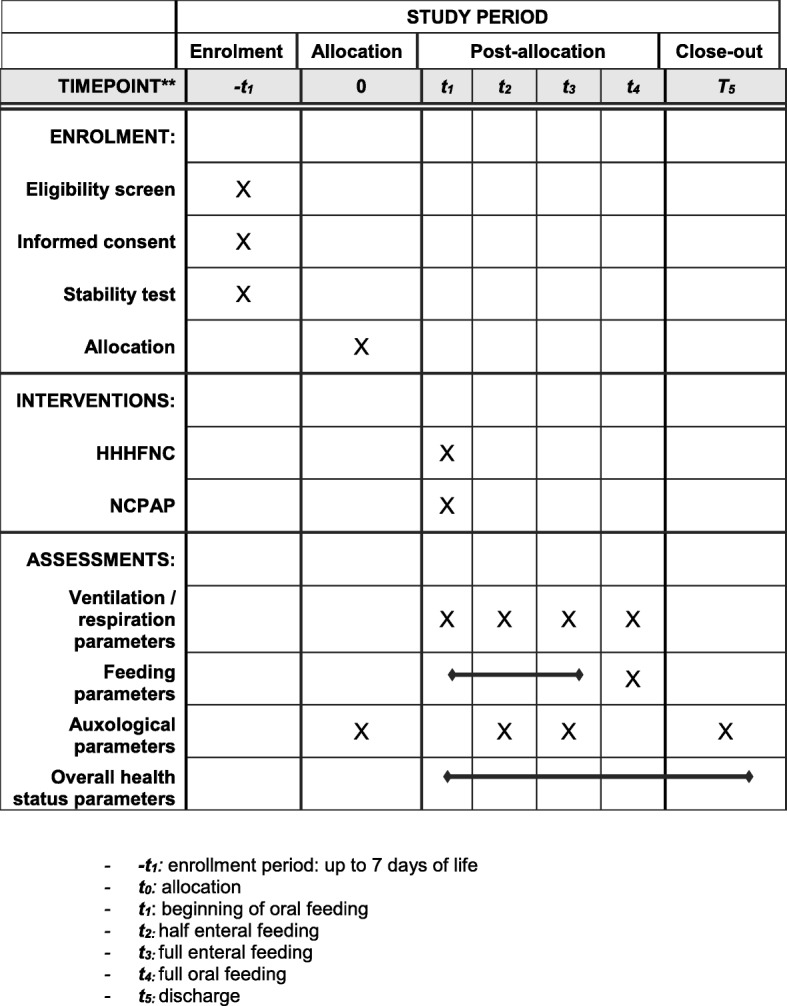
SPIRIT figure

The design of the study is outlined in Fig. 1. The Standard Protocol Items: Recommendations for Interventional Trials (SPIRIT) figure of enrollment, interventions, and assessments is shown in Fig. 2. The SPIRIT checklist is provided as Additional file 1.

### Statistical analysis and sample size

Time to reach FEF, the primary outcome, will be analyzed by Kaplan and Meier survival analysis according to the intention-to-treat principle. The two arms will be compared with the log-rank Test [36].

Regarding secondary outcomes, the time to reach half enteral feeding and time to reach full oral feeding will be estimated by Kaplan and Meier analysis, the failure of the respiratory support assigned at randomization will be compared using Fischer’s exact test, and the other secondary outcomes will be estimated using appropriate generalized linear models. This will be a single-blind trial where the blinded person will be the statistician.

Based on a population of infants with a gestational age < 30 weeks who are consecutively admitted to the NICUs of each research unit from January to June 2017 (mean time of FEF 19.6 days) and considering a ratio between the subjects of the two arms of 1:1, a sample size of at least 141 patients per arm has been calculated to observe a difference of 30% between the two arms (5.7 days).

An interim analysis is planned upon reaching the enrollment of half of the patients expected by the sample size calculation.

## Quality control and quality assurance procedures

### Compliance to protocol

Compliance will be defined as full adherence to protocol. Compliance with the protocol will be ensured by a number of procedures as described below.

### Site setup

Local principal investigators participated in preparatory meetings in which details on study protocol, non-invasive ventilation and feeding strategies, and data collection were accurately discussed. All units received detailed written instruction on web-based recording data, and to resolve possible difficulties it will be possible to contact the Clinical Trials Coordinating Unit (Dr. E Maggiora and Dr. SM Borgione).

### Safety

Safety endpoints will include incidence, severity, and causality of reported significant adverse events (SAEs). All SAEs will be followed until satisfactory resolution or until the investigator responsible for the care of the participant deems the event to be chronic or the patient to be stable. All expected and unexpected SAEs, whether or not they are attributable to the study intervention, will be reviewed by the local principal investigators to determine if there is a reasonable suspected causal relationship with the intervention. If the relationship is reasonable, SAEs will be reported to the chief investigators, who will then report them to the ethics committee and inform all other investigators to guarantee the safety of the participants.

## Discussion

The identification of the most suitable NRS technique for preterm infants with RDS and FI could reduce gastrointestinal complications, improve growth, and reduce hospital stay, thus improving quality of life of infants and their family and reducing health costs.

The evaluation of the timing of oral feeding could be useful in understanding the influence that NRS techniques have on the development of sucking-swallowing coordination.

A standard protocol for the suspension of feeding will be proposed along with a new clinical score to evaluate signs of FI. It may be useful to evaluate the influence, on clinical practice and on the time of achievement of FEF, of the application of a defined and shared method for the evaluation of feeding tolerance. The authors considered a difference of 30% in the time to reach FEF between the groups as the minimum needed to observe a clinically relevant effect. As a consequence, the sample of this study was set at 141 patients per arm.

The evaluation of the response to NCPAP and HHHFNC could clarify their efficacy as treatment for RDS in extremely preterm infants.

## Trial status

The protocol is version no. 1, 24 April 2018. The recruitment will begin after approval by the ethics committee of all research units and is expected to begin on 15 September 2018. The time expected to complete the recruitment is about 2 years.

## Additional file

Additional file 1: SPIRIT 2013 checklist: recommended items to address in a clinical trial protocol and related documents. (DOC 121 kb)

## Abbreviations

BPD: Bronchopulmonary dysplasia
FI: Feeding intolerance
HHHFNC: Heated humidified high-flow nasal cannula
NCPAP: Nasal continuous positive air pressure
NEC: Necrotizing enterocolitis
NICU: Neonatal intensive care unit
NRS: Non-invasive respiratory support
RDS: Respiratory distress syndrome
SAE: Significant adverse event
TC: Transcutaneous
